# Fine mapping of candidate effector genes for heart rate

**DOI:** 10.1007/s00439-024-02684-z

**Published:** 2024-07-06

**Authors:** Julia Ramírez, Stefan van Duijvenboden, William J. Young, Yutang Chen, Tania Usman, Michele Orini, Pier D. Lambiase, Andrew Tinker, Christopher G. Bell, Andrew P. Morris, Patricia B. Munroe

**Affiliations:** 1 Aragon Institute of Engineering Research, University of Zaragoza, Zaragoza, Spain; 2 Centro de Investigación Biomédica en Red – Bioingeniería, Biomateriales y Nanomedicina, Zaragoza, Spain; 3 William Harvey Research Institute, Barts and the London Faculty of Medicine and Dentistry, Queen Mary University of London, London, EC1M 6BQ UK; 4 Nuffield Department of Population Health, University of Oxford, Old Road Campus, Headington, Oxford, OX3 7LF UK; 5 Institute of Cardiovascular Science, University College London, London, UK; 6 Department of Environmental Systems Science, ETH Zurich, Zurich, Switzerland; 7 Barts Heart Centre, St Bartholomew’s Hospital, London, EC1A 7BE UK; 8 Kings College London, London, UK; 9 Khalifa University, Abu Dhabi, United Arab Emirates; 10 Barts Cardiovascular Biomedical Research Centre, National Institute of Health and Care Research, Queen Mary University of London, London, EC1M 6BQ UK; 11 Centre for Musculoskeletal Research, The University of Manchester, Manchester, UK; 12 National Institute of Health and Care Research, Manchester Biomedical Research Centre, Manchester University NHS Foundation Trust, Manchester Academic Health Science Centre, Manchester, UK

## Abstract

An elevated resting heart rate (RHR) is associated with increased cardiovascular mortality. Genome-wide association studies (GWAS) have identified > 350 loci. Uniquely, in this study we applied genetic fine-mapping leveraging tissue specific chromatin segmentation and colocalization analyses to identify causal variants and candidate effector genes for RHR. We used RHR GWAS summary statistics from 388,237 individuals of European ancestry from UK Biobank and performed fine mapping using publicly available genomic annotation datasets. High-confidence causal variants (accounting for > 75% posterior probability) were identified, and we collated candidate effector genes using a multi-omics approach that combined evidence from colocalisation with molecular quantitative trait loci (QTLs), and long-range chromatin interaction analyses. Finally, we performed druggability analyses to investigate drug repurposing opportunities. The fine mapping pipeline indicated 442 distinct RHR signals. For 90 signals, a single variant was identified as a high-confidence causal variant, of which 22 were annotated as missense. In trait-relevant tissues, 39 signals colocalised with cis-expression QTLs (eQTLs), 3 with cis-protein QTLs (pQTLs), and 75 had promoter interactions via Hi-C. In total, 262 candidate genes were highlighted (79% had promoter interactions, 15% had a colocalised eQTL, 8% had a missense variant and 1% had a colocalised pQTL), and, for the first time, enrichment in nervous system pathways. Druggability analyses highlighted *ACHE*, *CALCRL*, *MYT1* and *TDP1* as potential targets. Our genetic fine-mapping pipeline prioritised 262 candidate genes for RHR that warrant further investigation in functional studies, and we provide potential therapeutic targets to reduce RHR and cardiovascular mortality.

**Supplementary Information:**

The online version contains supplementary material available at 10.1007/s00439-024-02684-z.

## Introduction

An elevated resting heart rate (RHR) has been associated with an increased cardiovascular morbidity and mortality independent of traditional risk factors (Zhang et al. 2016). Reduction of heart rate using pharmacological intervention is an important component of therapy of a number of cardiovascular conditions including angina pectoris and heart failure. For example, direct inhibition of the pacemaker current with ivabradine reduces cardiovascular events in heart failure (Cargnoni et al. 2006), suggesting that RHR is a modifiable risk factor. However, the exact mechanisms linking RHR to risk are still not clear.

Genetics contributes to up to 20% of the interindividual variance in RHR (van de Vegte et al. 2023) and, at present, genome-wide association studies (GWAS) and exome-array wide association studies have identified > 350 loci for RHR explaining > 5% of this variance (den Hoed et al. 2013; Eijgelsheim et al. 2010; Eppinga et al. 2016; Guo et al. 2019; van de Vegte et al. 2023; van den Berg et al. 2017). The underlying effector genes remain unknown for most of these loci, which limits our understanding of the genetic and biological mechanisms of RHR. Utilising a fine-mapping approach could identify responsible effector genes and biological pathways explaining the mechanisms underlying RHR and its relation to cardiovascular risk, as well as novel therapeutic targets.

One promising avenue to improve prioritization of causal variants and candidate genes is the integration of GWAS with functional genomic information data to fine map GWAS loci. Previous studies have shown it can substantially improve the causal-variant resolution for risk loci for Type-2 diabetes (Mahajan et al. 2018) and blood pressure(van Duijvenboden et al. 2023). These studies leveraged European ancestry GWAS to avoid the calibration issues of fine-mapping across multi-ancestry meta-analysis GWAS (Kanai et al. 2022).

**Fig. 1:**
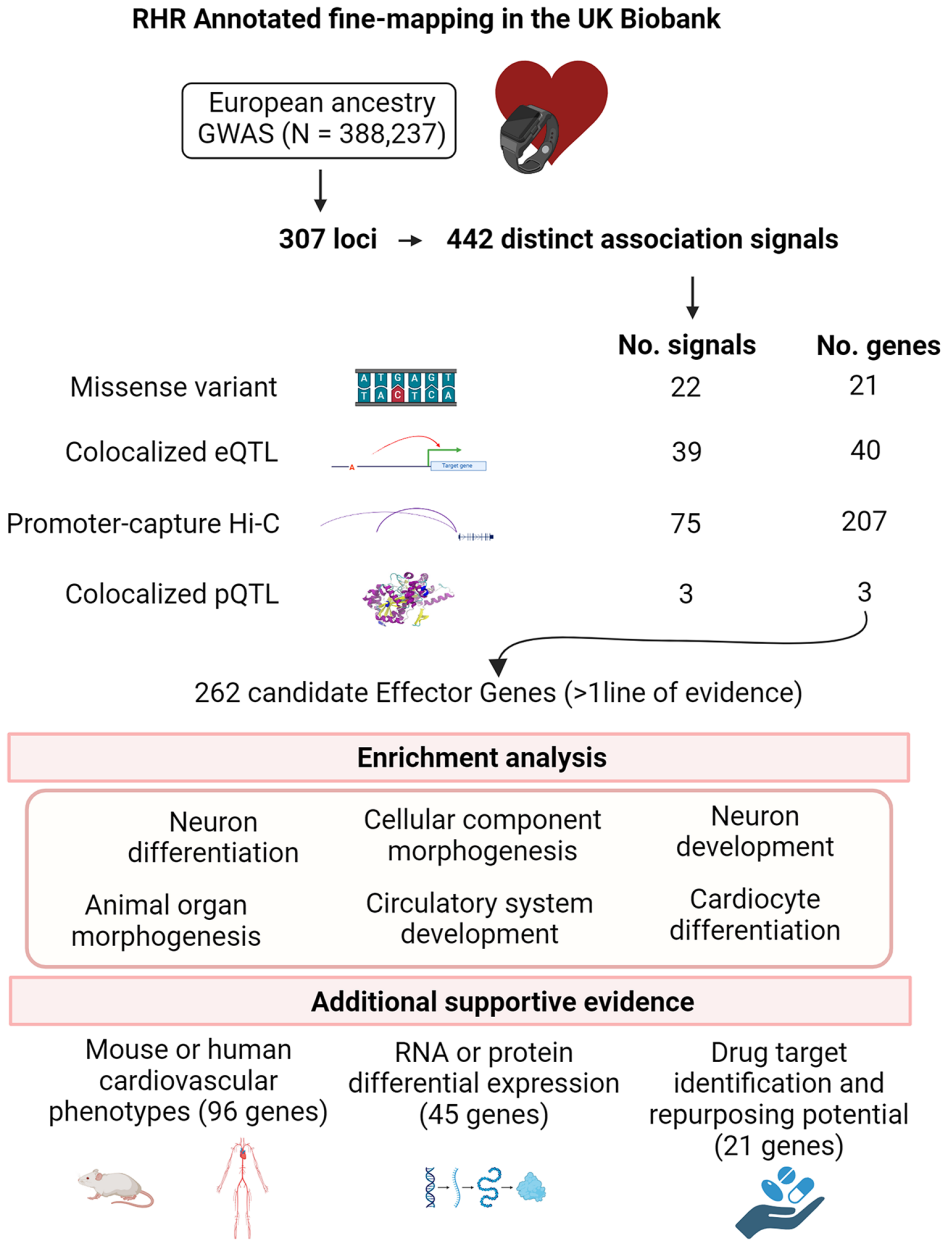
Overview of the study and summary of main findings. Created with BioRender.com. eQTL, expression quantitative locus; GWAS, genome-wide association study; Hi-C, long-range chromatin interaction; pQTL, protein quantitative locus; RHR, resting heart rate; RNA, ribosomal nucleic acid

In the present work, we performed an annotation-informed fine-mapping analysis using a European ancestry GWAS to identify causal variants and candidate effector genes for RHR. Prioritisation of candidate effector genes was based on evidence from functional annotation, colocalisation analyses with expression and protein quantitative trait loci (eQTLs and pQTLs) and promoter capture Hi-C interactions in relevant RHR tissues. We investigated the biological pathways of the prioritised effector genes. We also investigated additional evidence of support for effector genes from mouse and human phenotypes and differential expression. Finally, we assessed the potential of the prioritised effector genes for drug target identification and repurposing opportunities. An overview of the study is shown in Fig. 1.

## Methods

### Identification of distinct associations signals for fine-mapping

We conducted a fine-mapping analysis of RHR GWAS summary statistics of 388,237 individuals of European ancestry from UK Biobank (Mensah-Kane et al. 2021). The work was undertaken as part of UK Biobank application 8256. We initially defined loci as mapping 500 kb up- and down-stream of each lead SNV (at genome-wide significance, *P* < 5 × 10^− 8^). Where loci boundaries overlapped, they were combined as a single locus. We then performed approximate conditional analyses using GCTA-COJO(Yang et al. 2012) to detect distinct association signals at each locus using unrelated individuals with European ancestry as a reference for linkage disequilibrium (LD). Within each locus, variants attaining genome-wide significance (*P* < 5 × 10^− 8^) in the joint GCTA-COJO model were selected as index SNVs for distinct association signals.

### Enrichment of RHR associations for genomic annotations

We used fGWAS(Pickrell 2014) to identify genomic annotations from a total of 253 functional and regulatory annotations (via GENCODE and Roadmap Epigenomics)(Harrow et al. 2012; Kundaje et al. 2015) that were enriched for RHR association signals (Supplemental Methods). We then used an iterative approach to identify a joint model of enriched annotations using a forward-selection approach. At each iteration, we added the annotation to the joint fGWAS model that maximised the improvement in the penalised likelihood. We continued until no additional annotations improved the fit of the joint model (*P* < 0.00020, Bonferroni correction for 253 annotations).

### Fine-mapping distinct association signals for RHR

For each $j$th variant at the $i$th distinct signal, we first estimated its prior probability of causality using an annotation-informed prior model:

$${\gamma }_{j}=\text{e}\text{x}\text{p}\left[{\sum }_{k}{\widehat{\beta }}_{k}{z}_{jk}\right]$$,

where the summation is over the enriched annotations, ${\widehat{\beta }}_{k}$ is the estimated log-fold enrichment of the $k$th annotation from the final joint fGWAS model, and ${z}_{jk}$ is an indicator variable taking the value 1 if the $j$th variant maps to the $k$th annotation, and 0 otherwise. We then approximated the Bayes’ factor, ${\varLambda }_{ij}$, using the European ancestry summary statistics, as previously described(van Duijvenboden et al. 2023) (Supplemental Methods). Finally, we estimated the $j$th variant posterior probability of causality as ${\pi }_{ij}\propto {{\gamma }_{j}\varLambda }_{ij}$.

Next, we derived a 99% credible set for the $i$th distinct association signal by: (i) ranking all SNVs according to ${\pi }_{ij}$; and (ii) including ranked variants until their cumulative posterior probability attains or exceeds 99%(van Duijvenboden et al. 2023). The credible set would, then, include the minimum number of variants that jointly explained ≥ 99% of the posterior probability of driving the RHR association under the annotation-informed prior. We defined high-confidence causal variants as single variants from the credible sets accounting for more than 75% of the posterior probability.

### Functional annotation of variants

We used variant-effect predictor (VEP) analysis(McLaren et al. 2016) to annotate the high-confidence causal variants from the credible sets, and selected those annotated as missense variants.

### Colocalisation with gene expression data

We integrated genetic fine-mapping data with *cis*-eQTL in adrenal gland, artery, heart, nerve and brain tissues from the GTEx Consortium version 8 (tissue selection was informed by tissue enrichment analysis from prior GWAS(Eppinga et al. 2016) and biological mechanisms known to regulate RHR, Supplemental Methods). We first did a lookup of significant lead eQTL variants in the 99% credible sets. For each signal where we detected overlap, we formally assessed whether the annotation informed Bayes’ factor for the credible set variants of the corresponding signal colocalised with the eQTL results, as previously described(van Duijvenboden et al. 2023).

### Long-range chromatin interaction (Hi–C) analyses

We identified potential target genes of regulatory SNVs using long-range chromatin interaction (Hi–C) data from adrenal gland, aorta, left and right ventricles, hippocampus and cortex(Jung et al. 2019) - similar tissues as selected for eQTL analysis. Hi–C data was corrected for genomic biases and distance using the Hi–C Pro and Fit-Hi-C pipelines according to Schmitt et al(Schmitt et al. 2016). From the Hi–C data, we report the target genes with which these high regulatory potential SNVs interact (Supplemental Methods).

### Colocalisation with protein expression data

We additionally integrated genetic fine-mapping data with protein quantitative trait loci (*cis*-pQTL) in plasma(Ferkingstad et al. 2021). We performed the same Bayesian statistical procedure as for eQTL colocalisation to assess whether those signals for which a 99% credible set variant was the lead pQTL variant, colocalised with pQTL results.

### Prioritisation of candidate effector RHR genes

A full list of candidate effector genes for RHR was collated from the results of our fine-mapping pipeline and computational approaches, similar to our studies on blood pressure as reported recently(van Duijvenboden et al. 2023). A gene was indicated for a signal if there was support from a coding and high-confidence variant in the gene at the locus, or if the gene was indicated from eQTL, pQTL colocalization or promoter capture Hi-C analyses.

### Effector gene pathway analysis

We used the Gene2Function analysis tool in FUMA (v1.4.0) to perform gene set enrichment on the prioritised list of candidate genes, and to identify significantly associated Gene Ontology (GO) terms and pathways(Watanabe et al. 2017). Redundant GO terms were removed using the Reduce and Visualize Gene Ontology (REVIGO) web application(Supek et al. 2011). Dispensability cut off < 0.7 was used in this analysis to remove redundant terms.

### Additional evidence for effector genes from mouse and human phenotypes and differential expression

We collated additional information for each prioritised candidate gene using data from GeneCards (https://genealacart.genecards.org). This included evidence from mouse model phenotypes, from the Human Phenotype Ontology database, from differential RNA and protein expression of the candidate gene in the GTEx database in cardiovascular tissues (Supplemental Methods).

### Druggability of prioritised effector genes

To identify candidate druggable targets, a look-up was done of the prioritised list of candidate genes in a previously published database of the druggable genome (Finan et al. 2017). This database categorises gene targets into tiers according to whether they are existing targets of approved drugs or drugs under development (Tier 1), greater than 50% shared protein sequence identity to existing targets (Tier 2), and extracellular proteins or members of key druggable gene families not already in Tier 1 or Tier 2 (Tier 3). To identify opportunities for drug repurposing, a look-up of each candidate gene was performed for Tier 1 to identify targets of licensed medication using the KEGG drug database (Supplementary Methods). The open targets database was interrogated to identify disease associations with each gene to identify overlap that could indicate a promising drug target. To identify enrichment of candidate effector genes in clinical indication categories and potentially re-positional drugs, we utilised the Genome for REPositioning drugs (GREP) software. A pathway-set enrichment analysis was also performed using Gene2Drug to identify drug repositioning candidates. Using RHR significant GO biological processes as input, pathway expression profiles are created and ranked according to the p-value of their Kolmogorov-Smirnov statistic that is used to search for drugs that up-regulate or dysregulate most pathways in the set(Napolitano et al. 2017).

## Results

### Fine-mapping and genomic annotation reveals high-confidence causal variants

**Fig. 2:**
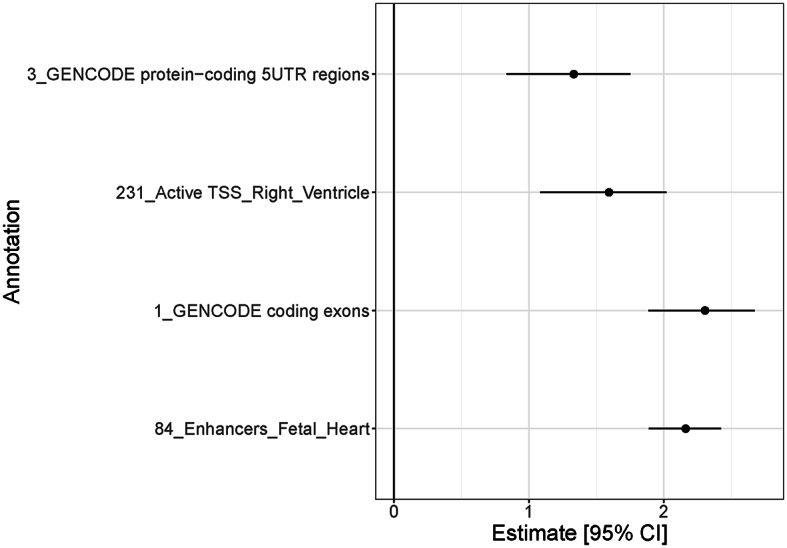
Results from genomic enrichment annotation for RHR. Estimate and 95% confidence interval of the log-fold enrichment at the most significant annotations for RHR, calculated using functional GWAS. UTR, untranslated region; TSS, transcription start site; RHR, resting heart rate

There were 307 genome-wide significant loci in the European GWAS performed by Mensah-Kane et al. (Mensah-Kane et al. 2021). We partitioned these loci into a total of 442 distinct association signals (Supplemental Table 1). We observed significant joint enrichment for RHR associations mapping to protein coding exons and 5’ UTRs, enhancers in the heart, and promoters in the right ventricle (Fig. 2, Supplemental Table 2).

**Fig. 3:**
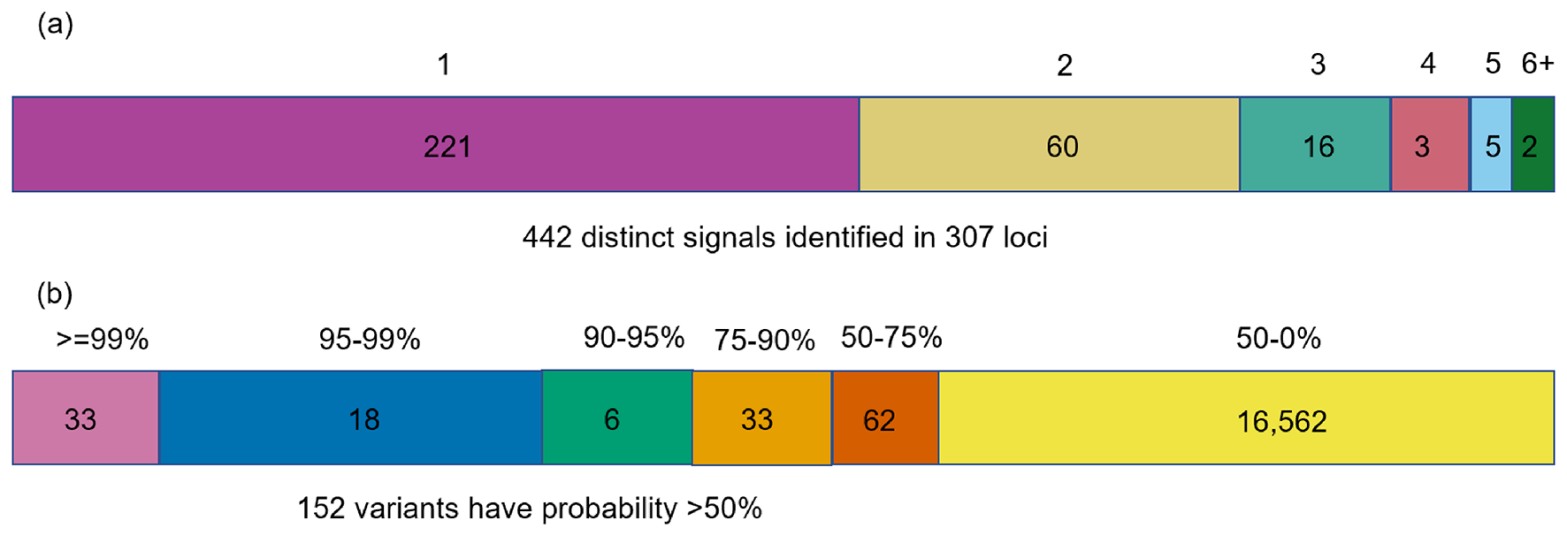
(**a**) Distinct RHR association signals. Summary of distinct association signals for RHR. A single signal at 221 genomic regions and at least two at 52. (**b**) Distribution of the posterior probability of causality of the variants in credible sets. RHR, resting heart rate

Using the enriched annotations, for each of the 442 distinct signals, we derived 99% credible sets of variants. The median 99% credible set size was 26 variants (Supplemental Table 1). For 90 (20.4%) RHR signals, a single SNV accounted for > 75% of the posterior probability of driving the RHR association under the annotation-informed prior, which we defined as “high-confidence” for causality (Fig. 3, Supplemental Table 3).

### Missense variants implicate causal genes

From the 90 high-confidence variants, 22 were missense variants in 21 genes (Table 1), of which seven were annotated as damaging and deleterious by PolyPhen and SIFT, respectively.

**Table 1:** High-confidence variants annotated as missense

Missense variant	Signal ID	Sentinel SNV	Canonical transcript	Gene	CHR	BP	Amino acid change	Polyphen	SIFT	PP	MAF
rs709209	1_1	rs709209	ENST00000377939.4	*RNF207*	1	6,278,414	p.Asn573Ser	Ben	Tol low conf	0.997	0.342
rs1260326	34_1	rs1260326	ENST00000264717.2	*GCKR*	2	27,730,940	p.Leu446Pro	Ben	Tol	0.994	0.396
rs3100246	35_1	rs3100246	ENST00000401605.1	*CLIP4*	2	29,383,256	p.Arg486Leu	Ben	Del	0.998	0.152
rs17362588	51_3	rs17362588	ENST00000343876.2	*CCDC141*	2	179,721,046	p.Arg379Trp	Prob Dam	Del	0.978	0.088
rs10497529	51_5	rs10497529	ENST00000420890.2	*CCDC141*	2	179,839,888	p.Ala141Val	Prob Dam	Del	1.000	0.037
rs4833772	96_1	rs4833772	ENST00000424958.1	*AC079341.1*	4	122,687,491	p.Asp98His	Unknown	-	0.790	0.488
rs28925904	97_1	rs28925904	ENST00000262995.4	*GAB1*	4	144,359,490	p.Pro311Leu	Poss dam	Del	0.990	0.025
rs2307111	106_1	rs2307111	ENST00000428202.2	*POC5*	5	75,003,678	p.His36Arg	Ben	Tol	1.000	0.395
rs12514461	108_1	rs12514461	ENST00000446378.2	*CMYA5*	5	79,041,057	p.Lys3583Glu	Poss dam	Tol	0.940	0.116
rs1015149	127_1	rs9471627	ENST00000230323.4	*TFEB*	6	41,658,889	p.His21Gln	-	-	0.803	0.459
rs80095409	147_1	rs150990106	ENST00000393561.1	*LAMB1*	7	107,600,211	p.Arg819Gly	Prob Dam	Del	0.709	0.014
rs6271	175_2	rs6271	ENST00000393056.2	*DBH*	9	136,522,274	p.Arg549Cys	Poss dam	Tol	0.990	0.074
rs7102584	205_3	rs7102584	ENST00000338350.4	*KCNJ5*	11	128,782,012	p.Gln282Glu	Ben	Tol	0.999	0.018
rs3184504	221_2	rs10774625	ENST00000538307.1	*SH2B3*	12	111,884,608	p.Trp60Arg	Ben	Del	0.701	0.481
rs12889267	230_1	rs12889267	ENST00000555038.1	*ARHGEF40*	14	21,542,766	p.Lys293Glu	Poss dam	Del	1.000	0.167
rs365990	231_1	rs365990	ENST00000356287.3	*MYH6*	14	23,861,811	p.Val1101Ala	Ben	Tol	1.000	0.370
rs5742915	243_4	rs5742915	ENST00000268058.3	*PML*	15	74,336,633	p.Phe645Leu	Ben	Tol	1.000	0.461
rs238238	258_1	rs238238	ENST00000323997.6	*ENO3*	17	4,856,376	p.Asn71Ser	Ben	Tol low conf	0.680	0.296
rs61735998	276_3	rs61735998	ENST00000590592.1	*FHOD3*	18	34,289,285	p.Val822Phe	Poss dam	Del	1.000	0.025
rs7412	290_1	rs190712692	ENST00000252486.4	*APOE*	19	45,412,079	p.Arg176Cys	Prob Dam	Del	0.810	0.081
rs17265513	297_1	rs17265513	ENST00000544979.2	*ZHX3*	20	39,832,628	p.Asn310Ser	Poss dam	Tol	0.979	0.199
rs148377517	298_1	rs148377517	ENST00000396825.3	*FITM2*	20	42,939,693	p.Met32Ile	Ben	Tol	1.000	0.015

CHR, chromosome; BP, base pair position in human genome build 37; sentinel SNV, lead variant in the signal; Missense.variant, high-confident variant in the signal annotated as missense; PP, posterior probability of the high-confidence variant driving the association with RHR; MAF, minor allele frequency of the missense variant; Polyphen and SIFT columns indicate the consequence prediction from each tool, respectively; Tol, tolerated consequence; Del, deleterious consequence; Ben, benign consequence; Prob Dam, probably damaging consequence; Poss dam, possibly damaging consequence; conf, confidence

Four variants were annotated as probably damaging and deleterious were in *CCDC141* (rs17362588, p.Arg379Trp and rs10497529, p.Ala141Val), *APOE (*rs7412, p.Arg176Cys), and *LAMB1* (rs80095409, p.Arg819Gly), Table 1). We found two missense variants in *CCDC141*. This gene is involved in axon guidance and cell adhesion and plays a critical role in radial migration and centrosomal function(Saengkaew et al. 2021). It has been identified by previous analyses for RHR(van den Berg et al. 2017), RHR dynamics(Ramírez et al. 2018; Verweij et al. 2018) and sick sinus syndrome(Thorolfsdottir et al. 2021). However, there are yet no functional studies investigating the mechanistic link of *CCDC141* and RHR. The *APOE* variant, rs7412, determines the *APOE2* isoform, which has been shown in both human and animal studies to be protective against Alzheimer’s Disease and is additionally associated with longevity independent of Alzheimer’s Disease (Shinohara et al. 2020). Previous studies suggest that laminins have important roles in human heart development and function(Haag et al. 1999). In particular for *LAMB1* (Laminin Subunit Beta 1) zebrafish embryos had mild morphogenetic defects and progressive cardiomegaly, as well as a limited heart size during cardiac development(Derrick et al. 2021).

Three missense variants were annotated as possibly damaging and deleterious by PolyPhen and SIFT: *FHOD3* (rs61735998, p.Val822Phe), *ARHGEF40* (rs12889267, p.Lys293Glu) and *GAB1* (rs28925904, p.Pro311Leu). The variants in *FHOD3* and *ARHGEF40* had a 100% posterior probability of driving the RHR signal. *FHOD3* is essential for myofibrillogenesis at an early stage of heart development(Kan-O et al. 2012) and has been identified as a causal gene for hypertrophic cardiomyopathy with related heart rate abnormalities in humans (Ochoa et al. 2018). *ARHGEF40* has previously been associated with all-cause mortality (Eppinga et al. 2016), but is less functionally characterised and encodes a protein similar to guanosine nucleotide exchange factors for Rho GTPases. Finally, *GAB1* is an adapter protein that plays a role in intracellular signalling cascades triggered by activated receptor-type kinases (Yousaf et al. 2018). Cardiac-specific *GAB1* knock-out in mice has been reported to lead to dilated cardiomyopathy associated with mitochondrial damage and cardiomyocyte apoptosis (Zhao et al. 2016).

### Genes identified using gene expression in disease relevant tissues

Convincing support for colocalization with gene expression in at least one tissue was identified for 39 distinct signals at 40 genes (Supplemental Table 4). A total of 21 signals (for 21 genes) colocalised at a single tissue, 18 at heart or arterial tissue and 3 at brain. There were no specific colocalisations in adrenal gland tissues (Supplemental Fig. 1). We observed 14 signals (for 14 genes) that colocalised at more than one tissue. For the 4 remaining signals, one signal indicated two genes (*AC009264.1* and *CHRM2*) in the heart left ventricle, there were two signals for one gene (*CPNE5*) in the heart left ventricle, and for the last signal two genes (*FLCN* and *PLD6*) were colocalised in multiple tissues (brain, heart and artery).

An interesting gene with heart-specific colocalisation is *PLEC*. *PLEC* knock out mouse models show right bundle branch block and abnormal heart morphology, and a missense variant in *PLEC* has been reported to increase risk of atrial fibrillation in humans(Thorolfsdottir et al. 2017).

There were a few genes with brain-specific colocalisations, including *NKX2-5*, *LEMD2* and *UCK1*. *NKX2-5*’s reported function is a regulator of cardiovascular development(Bruneau 2008), with mouse and zebrafish models showing abnormalities in heart rate, among other cardiovascular phenotypes (Table 2). However, the function of *NKX2-5* in the brain is not reported. There are *LEMD2* mouse and human models that include an arrhythmogenic cardiomyopathy phenotype(Gerull and Brodehl 2020). Finally, *UCK1* phosphorylates uridine and cytidine to uridine monophosphate and cytidine monophosphate (GeneCards), but there is no data indicating association with RHR or cardiovascular phenotypes.

**Table 2:** Twenty-three candidate genes for RHR with additional evidence of support from mouse or human phenotypes and RNA or protein differential expression

			Bioinformatics support				Gene
Signal ID	rsID	Index SNV	Missense high-confidence SNV	eQTL colocalisation	Hi.C gene	pQTL colocalisation	
28_1	rs12724121	1:236852282:A: T		*ACTN2(1)*			ACTN2*
51_3	rs17362588	2:179721046:G: A	*CCDC141*				*CCDC141*
67_1	NA	3:37551515:C: CT			*ITGA9(1)*		*ITGA9*
67_4	rs7372712	3:38686192:C: T			*SCN10A(1)*		SCN10A*
70_3	rs9819463	3:53672471:T: C			*CACNA1D(1)*		CACNA1D*
108_1	rs12514461	5:79041057:A: G	*CMYA5*				*CMYA5*
119_1	rs10071514	5:172669771:A: G		*NKX2-5(2)*			NKX2-5*
146_3	rs13244629	7:100509253:A: C		*ACHE(4)*			ACHE*
153_1	rs1424569	7:136569416:T: C		*CHRM2(1)*			CHRM2*
188_1	rs10787270	10:112459906:G: A			*RBM20(2)*		RBM20*
205_3	rs7102584	11:128782012:C: G	*KCNJ5*				KCNJ5*
221_2	rs10774625	12:111910219:A: G	*SH2B3*		*SH2B3(1)*		*SH2B3*
231_1	rs365990	14:23861811:A: G	*MYH6*				MYH6*
231_3	rs117526881	14:23908895:G: C			*IL25(1)*		*IL25*
254_1	rs8048448	16:30692208:T: C			*FBXL19(5)*		*FBXL19*
254_1	rs8048448	16:30692208:T: C			*YPEL3(2)*		*YPEL3*
273_1	rs4800401	18:20003625:C: T			*GATA6(3)*		GATA6*
276_3	rs61735998	18:34289285:G: T	*FHOD3*				FHOD3*
290_1	rs190712692	19:45425178:G: A			*CKM(1)*		*CKM*
296_1	rs6123471	20:36840156:T: C			*GHRH(1)*		*GHRH*
296_1	rs6123471	20:36840156:T: C			*TGM2(1)*		TGM2*
298_1	rs148377517	20:42939693:C: T	*FITM2*				*FITM2*
305_1	rs749085725	22:20098887:CT: C			*SCARF2(1)*		*SCARF2*

Signal.ID, distinct signal identifier, based on the locus number and signal number within the locus; rsID, rsID of the lead SNV in the signal; Index.SNV, chromosome: base pair in build 37:reference allele: other allele; SNV, single-nucleotide variant; eQTL, expression quantitative locus; Hi.C, long-range high-chromatin interaction; pQTL, protein quantitative locus. The number in brackets in the eQTL and Hi.C columns indicate the number of tissues at which we found support. *Indicates there is a mouse or human abnormal heart rate phenotype, as indicated in Supplementary Table 7

### Identification of genes using long-range chromatin interactions

Promoter interactions were identified for 75 distinct signals mapping to 207 genes at 66 loci (Supplemental Table 5). A total of 107 genes were indicated in a single tissue, of which 14 (13%) were in left or right ventricle, 83 (78%) in brain, and 10 (9%) in the adrenal gland. From the genes specifically indicated in heart tissue, *SCN10A* has been thoroughly characterised as a RHR modifier(Eppinga et al. 2016; Ramírez et al. 2018)Table 2) and *CASZ1* is involved in cardiac morphogenesis and development, and there are abnormal mouse phenotypes including congenital and structural cardiomyopathies(Spielmann et al. 2022). There are also some genes that do not have experimental support for cardiovascular traits, these include *CABLES1*, *BET1* and *SLC22A17*. *CABLES1* encodes a protein involved in regulation of the cell cycle through interactions with several cyclin-dependent kinases(Malumbres 2014). *BET1* encodes a golgi-associated membrane protein that participates in vesicular transport from the endoplasmic reticulum to the Golgi complex(Hay et al. 1996). *SLC22A17* is a cell surface receptor for *Lipocalin 2*, an antibacterial protein that acts by sequestering iron during bacterial infection and has recently been reported to be involved in various pathophysiological conditions in various organs and tissues, including the heart and brain(Lim et al. 2021).

Two genes indicated in brain tissue with a low (2a) regulome score include *CEP68*, and *CISD3 (*Supplemental Table 5**)**. *CEP68* has a mouse cardiovascular phenotype (increased heart weight), and variants at this locus have previously been associated with atrial fibrillation (Christophersen et al. 2017). *CISD3* may play a role in regulating electron transport and oxidative phosphorylation(Wiley et al. 2007), and diseases associated with this gene include Wolfram Syndrome, a disorder which is associated with childhood diabetes, optic atrophy and deafness (OMIM number 222,300).

### Genes identified using protein expression

We identified significant pQTLs for three genes, *GCKR*, *ENO3* and *MXRA7* (Supplemental Table 6). These three genes also had support from other analyses; missense annotation (*GCKR*), and eQTL analyses (*ENO3* and *MXRA7*). *GCKR* regulates glucokinase by forming an inactive complex with this enzyme. Postprandial triglyceridemia is an emerging risk factor for cardiovascular disease and *GCKR* gene polymorphisms affect postprandial lipemic response in a dietary intervention study(Shen et al. 2009). *ENO3* has demonstrated increased differential expression in the left ventricle in rats(Giusti et al. 2009). The role of *MXRA7* (matrix-remodeling-associated protein 7) in potentially modulating RHR and cardiovascular disease is less known; but it has previously been associated with a cardiorespiratory fitness polygenic risk score(Cai et al. 2023).

### Candidate gene prioritisation

From the complementary fine mapping and computational approaches, we prioritised a total of 262 candidate genes for RHR that had at least one line of evidence (Supplemental Table 7). This list includes 21 genes with high-confidence missense variants (Table 1), 40 genes with colocalised eQTLs (Supplemental Table 4), 207 genes with Hi-C interactions (Supplemental Table 5) and 3 genes with colocalised pQTLs (Supplemental Table 6).

### Biological pathways

To gain insights into the biological role of the 262 candidate genes for RHR, we performed gene-set enrichment analyses via FUMA (Methods). We found significant enrichment for 41 unique GO biological processes (Supplemental Table 8). The most significant biological processes included cellular component morphogenesis (*P* = 2.1 × 10^− 4^), neuron differentiation (*P* = 4.6 × 10^− 4^), and neuron development (*P* = 8.9 × 10^− 4^).

### Additional functional evidence for RHR candidate effector genes

To explore the function of the 262 prioritised candidate genes, additional evidence for each candidate effector gene was assessed using mouse model data, human cardiovascular phenotypes and assessing differential expression of RNA/protein in cardiovascular tissues. We observed 74 of the 262 prioritised candidate genes (28.2%) to have support from mouse model data and 45 (17.2%) from human cardiovascular phenotypes (96 genes had support from mouse or human cardiovascular phenotypes). We also found 45 candidate genes (4.2%) had support from RNA or protein differential expression. In total, 23 candidate genes (9.2%) had additional functional evidence from both phenotypes and differential expression, and 12 of these 23 genes directly showed an abnormal heart rate phenotype in mouse or human experimental models (Table 2, Supplemental Table 7).

### Drug target identification and repositioning opportunities

We found 21 of the 262 candidate effector genes were existing targets of small molecules or biotherapeutics and clinical drug candidates (Tier 1, Supplemental Table 9). Of these, 5 were among the 23 candidate genes with additional functional evidence, *SCN10A*, *CACNA1D*, *ACHE*, *CHRM2* and *MYH6* (all directly liked to RHR through abnormal mouse or human phenotypes). *CACNA1D*, *MYH6* and *SCN10A* had a cardiovascular disease as their top disease association (sinoatrial node dysfunction, hypertrophic cardiomyopathy and AF, respectively). *CHRM2* and *CACNA1D* are established targets of drugs with an intention to either increase (atropine) or decrease (Diltiazem, Verapamil) RHR. In the remaining genes, the cardiovascular system was not implicated as top disease association, suggesting potential drug repurposing. For example, *ACHE* is a target of drugs for Alzheimer’s disease, including Donepezil and Galantamine, which cause bradycardia as a side effect.

The 262 candidate effector genes were enriched for gene-sets targeted by drugs for the nervous system, anaesthetics and gastrointestinal disorders (Supplemental Table 10). Pathway-directed investigation identified 48 drugs with support that dysregulate the GO biological processes either by up or down-regulation, including Isoprenaline and Atenolol (Supplemental Table 11). Of the top 10 drugs, Buspirone (5-Hydroxytryptamine 1 A receptor antagonist)(Osei-Owusu and Scrogin 2004) and Oxybuprocaine (anaesthetic) (Hung et al. 2010) have experimental evidence for an effect on RHR but are not current drug targets for cardiovascular diseases. The compound 2,6-dimethylpiperidine has previously demonstrated antiarrhythmic activity in a dog model however to the best of our knowledge, has not undergone further investigation (Hoefle et al. 1991).

## Discussion

In the present work, we employed a functionally informed statistical framework to advance from initial broadly associated genomic regions to the prioritisation of 262 candidate effector genes for RHR. From these, 23 had additional functional evidence and 21 were existing drug targets.

We used a functionally informed fine-mapping approach to specify plausible causal variants and 262 candidate genes for RHR using biologically interpretable annotations that were identified without prior knowledge of the trait. A recent paper by Van de Vegte et al., used a scoring system for gene prioritisation based on four criteria: proximity, coding, eQTL and evidence from Data-driven Expression Prioritized Integration for Complex Traits (DEPICT). They highlighted a lower number of effector genes, 39 genes with high level of support, *PHACTR2*, *ENO3* and *SENP2* being their top effector genes(van de Vegte et al. 2023). A direct comparison with our results is difficult because of methodological differences. First, our pipeline ranks variants based on annotation-informed posterior probability of causality, instead of *P*-values. Second, we included evidence from Hi-C interactions (79% of candidate effector genes had Hi-C interaction support). Third, our study focused on individuals with European ancestry, whereas they conducted a multi-ancestry approach, hence reporting additional loci from their GWAS, therefore the number of starting signals is different. However, looking at their 39 prioritised genes, we found 32 of the genomic regions where these genes mapped to were genome-wide significant in our European GWAS, demonstrating positional overlap (Supplemental Table 12). At these 32 signals, our fine-mapping pipeline indicated 18 had a high-confidence variant and were, thus, considered for candidate effector gene prioritization. Our methodology provided support for 12 genes indicated by the 18 signals (31% of the genes prioritised by van de Vegte et al.), including *ENO3*, and 5 of these 12 (42%) were in our list of 23 genes with additional evidence (*CCDC141*, *CMYA5*, *KCNJ5*, *MYH6* and *FHOD3*, Supplemental Table 12). At the remaining 6 signals, our methodology prioritised a different effector gene. The comparison between studies highlights that our annotation-informed fine-mapping approach, which utilises numerous layers of multi-omics data following the identification of signals with high-confidence causal variants, along with validating previously reported effector genes, has prioritised important candidate genes for RHR for the first time.

Previous studies have reported enrichment of associations in pathways involved in cardiac tissue development, muscle cell differentiation and pro-arrhythmic pathways for RHR(den Hoed et al. 2013; van de Vegte et al. 2023; van den Berg et al. 2017). Experimental studies have generally focused on providing functional evidence for RHR genes involved in the cardiovascular system(Liaqat et al. 2019). However, our enrichment results provide, for the first time, support for nervous system pathways, a role that is supported by existing knowledge for autonomic regulation of heart rate. The genes involved in these pathways, therefore, warrant further investigation in functional studies.

Twenty-nine of the 41 (> 50%) of the significant biological pathways identified using the 262 prioritised candidate genes implicate at least one of the 23 candidate genes with additional functional evidence. We highlight two of these 23 not previously prioritised as candidate genes for RHR, *CACNA1D* and *RBM20*. *CACNA1D*, which was identified in this work as an existing target of calcium channel blockers, is present in the membrane of most excitable cells and mediates calcium influx in response to depolarization(Fourbon et al. 2017). Associated diseases include sinoatrial node dysfunction and deafness(Liaqat et al. 2019). *RBM20* acts as a regulator of mRNA splicing of a subset of genes encoding key structural proteins involved in cardiac development, such as *TTN*, *CACNA1C*, *CAMK2D* or *PDLIM5/ENH*(Vieira-Vieira et al. 2022). Mutations in this gene have been associated with familial dilated cardiomyopathy(Hoogenhof et al. 2018).

Druggability analyses highlighted three candidate genes as top gene targets for cardiovascular disease and four for a neurological disease with repurposing potential, *ACHE*, *CALCRL*, *MYT1* and *TDP1*. *ACHE* is a target of drugs for Alzheimer’s disease including Donepezil and Galantamine, which cause bradycardia as a side effect. *CALCRL* is a target of drugs for migraine disorder. It is a receptor for adrenomedullin, together with *RAMP2*(Mackie et al. 2018). One of the reported mouse phenotypes is differences in heart rate of heterozygous *CALCRL* female and male mice(Pawlak et al. 2017). *MYT1* is a drug target for autism spectrum disorder and is less characterised, it binds to the promoter regions of proteolipid proteins of the central nervous system and plays a role in the developing nervous system(Lee et al. 2019). Finally, *TDP1* is a drug target for spinocerebellar ataxia type 1 with axonal neuropathy(Hirano et al. 2007), which correlates with cardiac autonomic dysfunction, predominantly parasympathetic(Pradhan et al. 2008). Future work should evaluate the causal link between these drug targets and RHR using Mendelian Randomisation analyses, as done recently(Schmidt et al. 2020).

We found limited overlap across the different lines of evidence for each candidate gene (Supplementary Table 7), as previously described(Gazal et al. 2022). The use of cis-eQTL analyses for candidate gene prioritisation has been demonstrated to have a high precision but a lower recall, whereas promoter-capture Hi-C data add specificity, resulting in a complementary approach. Nevertheless, the strong benefit of including Hi-C data is that it provides evidence of tissue-specific physical 3D contact between the candidate cis-regulatory element and a specific protein coding gene’s promoter. Furthermore, we observed that the Hi-C analysis was the line of evidence contributing most of the genes. This can be supported by a recent re-evaluation of the a priori functional likelihood of eQTLs, due to their skew towards promoter loci and large effect sizes(Mostafavi et al. 2023).

There are some limitations to our study, firstly the lack of population diversity in our analyses. The annotation-informed fine mapping analyses were performed in a European ancestry GWAS from UK Biobank despite larger meta-analyses including other ancestries being available(van de Vegte et al. 2023). The reason of this was to avoid the calibration issues of meta-analysis fine-mapping and the heterogeneities and noise in phenotyping found in large meta-analyses(Kanai et al. 2022). An additional weakness is that whilst benefitting from dense genotyping and imputation of common variants, this is not exhaustive in capturing all the potential phenotypically associated genetic variation within each locus. This will miss the possible impact of rare variants, as well as any poorly tagged larger variant (copy number variants, short tandem repeats, inversions, etc.). Finally, we used an established tool, fGWAS(Pickrell 2014), to perform a joint analysis of functional genomic and GWAS data, as done by other studies(Jagadeesh et al. 2022; Liu and Montgomery 2020), followed by a previously reported methodology(Mahajan et al. 2018; Morris et al. 2019; van Duijvenboden et al. 2023) as our fine-mapping approach. Other functionally informed fine-mapping tools, like PolyFun(Weissbrod et al. 2020), could have alternatively been used.

In conclusion, we have prioritised 262 candidate genes using annotation-informed fine mapping and implicated, along with previously observed enriched tissues, nervous system pathways for the first time. Our findings inform further investigations to improve the functional understanding of the biology underlying RHR and may enable novel preventive and therapeutic opportunities.

## Electronic supplementary material

Below is the link to the electronic supplementary material.

Supplementary Material 1

Supplementary Material 2

## Data Availability

The data generated during the analyses performed in this study can be found in the main and supplementary material.
