# Supplementary material for: Fine mapping of candidate effector genes for heart rate
Source: Hum Genet. 2024 Jul 6;143(9-10):1207–21. doi: 10.1007/s00439-024-02684-z (PMC11485034; doi:10.1007/s00439-024-02684-z)
Supplement: Supplementary file 2 — Supplementary Material 2 [file 439_2024_2684_MOESM2_ESM.docx]

**Supplementary Methods**

*Enrichment of RHR associations for genomic annotations*

Previous studies have demonstrated that improved localisation of causal variants driving association signals for complex human traits can be attained by integration of genetic fine-mapping data with genomic annotation(Schaid et al. 2018; van Duijvenboden et al. 2023). We used fGWAS(Pickrell 2014) to identify genomic annotations enriched for RHR association signals. We considered a total of 253 functional and regulatory annotations derived from: (i) genic regions (protein coding exons, 3’ UTRs and 5’ UTRs) as defined by the GENCODE Project(Harrow et al. 2012); and (ii) chromatin state predictions of promoters and enhancers across 125 tissues from the Roadmap Epigenome Consortium(Kundaje et al. 2015) via Epilogos (<http://compbio.mit.edu/epilogos/>). We then used an iterative approach to identify a joint model of enriched annotations using a forward-selection approach. At each iteration, we added the annotation to the joint fGWAS model that maximised the improvement in the penalised likelihood. We continued until no additional annotations improved the fit of the joint model (*P* < 0.00020, Bonferroni correction for 253 annotations).

*Fine-mapping distinct association signals for RHR*

We first approximated the Bayes’ factor (BF), $\Lambda_{ij}$, in favour of association of the $j$th SNV at the $i$th distinct association signal using the European ancestry summary statistics. Specifically,

$\Lambda_{ij}=\exp\left[ \frac{D_{ij}-\ln K_{ij}}{2} \right]$,

where $D_{ij}={b_{ij}^{2}}/{v_{ij}}$, and $b_{ij}$ and $v_{ij}$ are the allelic log-OR and corresponding variance, respectively, across the GWAS (here $K_{ij}=1$)(Kass and Raftery 1995). At loci with a single association signal, $b_{ij}$ and $v_{ij}$ were taken from the unconditional meta-analysis. However, for loci with multiple association signals, $b_{ij}$ and $v_{ij}$ were taken from the joint GCTA-COJO model, conditioning on the index SNVs for all other signals at the locus. The posterior probability for the $j$th SNV at the $i$th distinct signal, was then given by $\pi_{ij}\propto{\gamma_{j}\Lambda}_{ij}$, where $\gamma_{j}$ is the relative prior probability of causality for the $j$th SNV. We considered an annotation-informed prior model, for which

$\gamma_{j}=\exp\left[ \sum_{k} \hat{\beta}_{k}z_{jk} \right]$,

where the summation is over the enriched annotations, $\hat{\beta}_{k}$ is the estimated log-fold enrichment of the $k$th annotation from the final joint fGWAS model, and $z_{jk}$ is an indicator variable taking the value 1 if the $j$th SNV maps to the $k$th annotation, and 0 otherwise. Finally, we derived a 99% credible set(Maller et al. 2012) for the $i$th distinct association signal by: (i) ranking all SNVs according to their posterior probability $\pi_{ij}$; and (ii) including ranked SNVs until their cumulative posterior probability attains or exceeds 99%.

*Colocalisation with gene expression data*

We performed a Bayesian statistical procedure to assess whether our annotation-informed GWAS fine-mapping colocalized with eQTL signals. We integrated genetic fine-mapping data with expression quantitative trait loci (*cis*-eQTL) in adrenal gland, artery, heart, nerve and brain tissues from the GTEx Consortium version 8(Aguet et al. 2020). The tissue selection was informed by tissue enrichment analysis from prior GWAS (artery, heart and adrenal gland(Eppinga et al. 2016)) and biological mechanisms known to regulate RHR (nerve, and brain). We first did a lookup of significant lead eQTL variants in the 99% credible sets. For each signal where we detected overlap, we formally assessed whether the annotation informed Bayes’ factor for the credible set variants of the corresponding signal colocalised with the eQTL results, as previously described(van Duijvenboden et al. 2023). We undertook colocalization by using the annotation-informed BF with the Coloc software package in R(Giambartolomei et al. 2014).

*Long-range chromatin interaction (Hi–C) analyses*

We identified potential target genes of regulatory SNVs using long-range chromatin interaction (Hi–C) data from adrenal gland, aorta, left and right ventricles, hippocampus and cortex(Jung et al. 2019), similar tissues as selected for eQTL analysis. Hi–C data was corrected for genomic biases and distance using the Hi–C Pro and Fit-Hi-C pipelines according to Schmitt et al(Schmitt et al. 2016). We followed a similar procedure as in the eQTL colocalization analysis. We first identified signals where potential regulatory SNVs (RegulomeDB score ≤2) were in the 99% credible set. Then, from the promoter centred Hi–C data, we reported the effector target genes with which these high regulatory potential SNVs interact.

*Effector gene pathway analysis*

We used the Gene2Function analysis tool in FUMA (v1.4.0) to perform gene set enrichment on the prioritised list of candidate genes, and to identify significantly associated Gene Ontology (GO) terms and pathways(Watanabe et al. 2017). Redundant GO terms were removed using the Reduce and Visualize Gene Ontology (REVIGO) web application(Supek et al. 2011). REVIGO uses a hierarchical clustering method to remove highly similar terms, incorporating enrichment P-values in the selection process. Default settings (dispensability cut off <0.7) were used in this analysis.

*Additional evidence for effector genes from mouse and human phenotypes and differential expression*

We collated additional information for each prioritised candidate gene using data from GeneCards (https://genealacart.genecards.org). This included the following: 1) a mouse model from Mouse Genome Informatics which has a cardiovascular phenotype. 2) A cardiovascular or vascular phenotype described for the candidate gene in the Human Phenotype Ontology database. 3) Differential RNA expression of the candidate gene in the GTEx database in cardiovascular or vascular tissues, only genes with fold changes >4 in a tissue were selected. 4) Differential protein expression of the candidate gene based on 69 integrated normal proteomics datasets in HIPED (the Human Integrated Protein Expression Database). Genes with a fold change value of >6 and protein abundance value of >0.1 PPM in an anatomical were selected.

*Druggability of prioritised effector genes*

To identify candidate druggable targets, a look-up was performed in a previously published database of the druggable genome developed by Finan et al(Finan et al. 2017). This list contains protein-coding genes categorised into three tiers: Tier 1 are targets of approved drugs and some drugs in clinical development, including targets of small molecules and biotherapeutics; Tier 2 are proteins closely related to drug targets or associated with drug-like compounds (≥50% shared protein sequence identity); Tier 3 includes extracellular proteins and members of key drug target families in Tier 1 (e.g., G protein-coupled receptors). To identify potential opportunities for drug repurposing, a look-up of each BP candidate gene was performed in Tier 1 to identify existing drug targets (https://www.genome.jp/kegg/genes.html). Primary targets of antihypertensives were also identified using the KEGG drug database (https://www.genome.jp/kegg/drug/). The open targets database was subsequently interrogated to identify disease associations with each gene, to identify potential overlap that could indicate promising drug targets. Target, drug and disease association data was downloaded from the platform (https://platform.opentargets.org/downloads). Open targets calculates association scores to capture the data type (e.g., gene level) and source, to aggregate evidence for an association, by calculating the harmonic sum using a weighted vector of data source scores. This sum is divided by the maximum theoretical value, resulting in a score between 0 and 1. To identify enrichment of candidate effector genes in clinical indication categories and potentially re-positional drugs, we utilised the Genome for REPositioning drugs (GREP) software(Finan et al. 2017). GREP performs a series of Fisher’s exact tests, to identify enrichment of a gene-set in genes targeted by a drug in a clinical indication category (Anatomical Therapeutic Chemical Classification System [ATC] or ICD10 codes.

**References**

Aguet F, Anand S, Ardlie KG, Gabriel S, Getz GA, Graubert A, Hadley K, Handsaker RE, Huang KH, Kashin S, Li X, MacArthur DG, Meier SR, Nedzel JL, Nguyen DT, Segrè AV, Todres E, Balliu B, Barbeira AN, Battle A, Bonazzola R, Brown A, Brown CD, Castel SE, Conrad DF, Cotter DJ, Cox N, Das S, de Goede OM, Dermitzakis ET, Einson J, Engelhardt BE, Eskin E, Eulalio TY, Ferraro NM, Flynn ED, Fresard L, Gamazon ER, Garrido-Martín D, Gay NR, Gloudemans MJ, Guigó R, Hame AR, He Y, Hoffman PJ, Hormozdiari F, Hou L, Im HK, Jo B, Kasela S, Kellis M, Kim-Hellmuth S, Kwong A, Lappalainen T, Li X, Liang Y, Mangul S, Mohammadi P, Montgomery SB, Muñoz-Aguirre M, Nachun DC, Nobel AB, Oliva M, Park Y, Park Y, Parsana P, Rao AS, Reverter F, Rouhana JM, Sabatti C, Saha A, Stephens M, Stranger BE, Strober BJ, Teran NA, Viñuela A, Wang G, Wen X, Wright F, Wucher V, Zou Y, Ferreira PG, Li G, Melé M, Yeger-Lotem E, Barcus ME, Bradbury D, Krubit T, McLean JA, Qi L, Robinson K, Roche NV, Smith AM, Sobin L, Tabor DE, Undale A, Bridge J, Brigham LE, Foster BA, Gillard BM, et al. (2020) The GTEx Consortium atlas of genetic regulatory effects across human tissues. Science 369: 1318-1330. doi: doi:10.1126/science.aaz1776

Eppinga RN, Hagemeijer Y, Burgess S, Hinds DA, Stefansson K, Gudbjartsson DF, van Veldhuisen DJ, Munroe PB, Verweij N, van der Harst P (2016) Identification of genomic loci associated with resting heart rate and shared genetic predictors with all-cause mortality. Nature Genetics 48: 1557-1563. doi: 10.1038/ng.3708

Finan C, Gaulton A, Kruger FA, Lumbers RT, Shah T, Engmann J, Galver L, Kelley R, Karlsson A, Santos R, Overington JP, Hingorani AD, Casas JP (2017) The druggable genome and support for target identification and validation in drug development. Science Translational Medicine 9: eaag1166. doi: doi:10.1126/scitranslmed.aag1166

Giambartolomei C, Vukcevic D, Schadt EE, Franke L, Hingorani AD, Wallace C, Plagnol V (2014) Bayesian Test for Colocalisation between Pairs of Genetic Association Studies Using Summary Statistics. PLOS Genetics 10: e1004383. doi: 10.1371/journal.pgen.1004383

Harrow J, Frankish A, Gonzalez JM, Tapanari E, Diekhans M, Kokocinski F, Aken BL, Barrell D, Zadissa A, Searle S, Barnes I, Bignell A, Boychenko V, Hunt T, Kay M, Mukherjee G, Rajan J, Despacio-Reyes G, Saunders G, Steward C, Harte R, Lin M, Howald C, Tanzer A, Derrien T, Chrast J, Walters N, Balasubramanian S, Pei B, Tress M, Rodriguez JM, Ezkurdia I, van Baren J, Brent M, Haussler D, Kellis M, Valencia A, Reymond A, Gerstein M, Guigó R, Hubbard TJ (2012) GENCODE: The reference human genome annotation for The ENCODE Project. Genome Research 22: 1760-1774. doi: 10.1101/gr.135350.111

Jung I, Schmitt A, Diao Y, Lee AJ, Liu T, Yang D, Tan C, Eom J, Chan M, Chee S, Chiang Z, Kim C, Masliah E, Barr CL, Li B, Kuan S, Kim D, Ren B (2019) A compendium of promoter-centered long-range chromatin interactions in the human genome. Nature Genetics 51: 1442-1449. doi: 10.1038/s41588-019-0494-8

Kass RE, Raftery AE (1995) Bayes Factors. Journal of the American Statistical Association 90: 773-795. doi: 10.1080/01621459.1995.10476572

Kundaje A, Meuleman W, Ernst J, Bilenky M, Yen A, Heravi-Moussavi A, Kheradpour P, Zhang Z, Wang J, Ziller MJ, Amin V, Whitaker JW, Schultz MD, Ward LD, Sarkar A, Quon G, Sandstrom RS, Eaton ML, Wu Y-C, Pfenning A, Wang X, ClaussnitzerYaping Liu M, Coarfa C, Alan Harris R, Shoresh N, Epstein CB, Gjoneska E, Leung D, Xie W, David Hawkins R, Lister R, Hong C, Gascard P, Mungall AJ, Moore R, Chuah E, Tam A, Canfield TK, Scott Hansen R, Kaul R, Sabo PJ, Bansal MS, Carles A, Dixon JR, Farh K-H, Feizi S, Karlic R, Kim A-R, Kulkarni A, Li D, Lowdon R, Elliott G, Mercer TR, Neph SJ, Onuchic V, Polak P, Rajagopal N, Ray P, Sallari RC, Siebenthall KT, Sinnott-Armstrong NA, Stevens M, Thurman RE, Wu J, Zhang B, Zhou X, Abdennur N, Adli M, Akerman M, Barrera L, Antosiewicz-Bourget J, Ballinger T, Barnes MJ, Bates D, Bell RJA, Bennett DA, Bianco K, Bock C, Boyle P, Brinchmann J, Caballero-Campo P, Camahort R, Carrasco-Alfonso MJ, Charnecki T, Chen H, Chen Z, Cheng JB, Cho S, Chu A, Chung W-Y, Cowan C, Athena Deng Q, Deshpande V, Diegel M, Ding B, Durham T, Echipare L, Edsall L, Flowers D, Genbacev-Krtolica O, et al. (2015) Integrative analysis of 111 reference human epigenomes. Nature 518: 317-330. doi: 10.1038/nature14248

Maller JB, McVean G, Byrnes J, Vukcevic D, Palin K, Su Z, Howson JMM, Auton A, Myers S, Morris A, Pirinen M, Brown MA, Burton PR, Caulfield MJ, Compston A, Farrall M, Hall AS, Hattersley AT, Hill AVS, Mathew CG, Pembrey M, Satsangi J, Stratton MR, Worthington J, Craddock N, Hurles M, Ouwehand W, Parkes M, Rahman N, Duncanson A, Todd JA, Kwiatkowski DP, Samani NJ, Gough SCL, McCarthy MI, Deloukas P, Donnelly P, The Wellcome Trust Case Control C (2012) Bayesian refinement of association signals for 14 loci in 3 common diseases. Nature Genetics 44: 1294-1301. doi: 10.1038/ng.2435

Pickrell Joseph K (2014) Joint Analysis of Functional Genomic Data and Genome-wide Association Studies of 18 Human Traits. The American Journal of Human Genetics 94: 559-573. doi: 10.1016/j.ajhg.2014.03.004

Schaid DJ, Chen W, Larson NB (2018) From genome-wide associations to candidate causal variants by statistical fine-mapping. Nature Reviews Genetics 19: 491-504. doi: 10.1038/s41576-018-0016-z

Schmitt Anthony D, Hu M, Jung I, Xu Z, Qiu Y, Tan Catherine L, Li Y, Lin S, Lin Y, Barr Cathy L, Ren B (2016) A Compendium of Chromatin Contact Maps Reveals Spatially Active Regions in the Human Genome. Cell Reports 17: 2042-2059. doi: 10.1016/j.celrep.2016.10.061

Supek F, Bošnjak M, Škunca N, Šmuc T (2011) REVIGO Summarizes and Visualizes Long Lists of Gene Ontology Terms. PLOS ONE 6: e21800. doi: 10.1371/journal.pone.0021800

van Duijvenboden S, Ramírez J, Young WJ, Olczak KJ, Ahmed F, Alhammadi MJAY, Bell CG, Morris AP, Munroe PB (2023) Integration of genetic fine-mapping and multi-omics data reveals candidate effector genes for hypertension. bioRxiv: 2023.01.26.525702. doi: 10.1101/2023.01.26.525702

Watanabe K, Taskesen E, van Bochoven A, Posthuma D (2017) Functional mapping and annotation of genetic associations with FUMA. Nature Communications 8: 1826. doi: 10.1038/s41467-017-01261-5

**Supplementary Figures**

*KANK2*

*RNF207*

*SGIP1*

*ACTN2*

*LINC01814*

*CALCRL*

*B3GNT7*

*ATG4B*

*THRB*

*GNB4*

*PP12613*

*HAND2-AS1*

*NKX2-5*

*LEMD2*

*CPNE5*

*MED20*

*TCF21*

*JAZF1*

*MLXIPL*

*ACHE*

*AC009264.1*

*CHRM2*

*PLEC*

*SPAAR*

*UCK1*

*SVIL*

*AGAP5*

*ROM1*

*C11orf45*

*SSPN*

*RP11-6O2.3*

*CCDC154*

*CDH11*

*FLCN*

*PLD6*

*LRRC37A*

*SDK2*

*FBF1*

*MXRA7*

*RP11-1055B8.3*

**Heart/Artery tissue**

**Brain tissue**

**Adrenal tissue**

**Supplementary Figure 1:** Overlap of eQTL colocalization results across heart/artery, brain and adrenal tissues.
